# The Heat Conductivity Properties of Hemp–Lime Composite Material Used in Single-Family Buildings

**DOI:** 10.3390/ma13041011

**Published:** 2020-02-24

**Authors:** Sławomir Pochwała, Damian Makiola, Stanisław Anweiler, Michał Böhm

**Affiliations:** 1 Department of Thermal Engineering and Industrial Facilities, Faculty of Mechanical Engineering, Opole University of Technology, Ulica Prószkowska 76, 45-758 Opole, Poland; dmakiola@gmail.com; 2 Department of Environmental Engineering, Faculty of Mechanical Engineering, Opole University of Technology, Ulica Prószkowska 76, 45-758 Opole, Poland; s.anweiler@po.edu.pl; 3 Department of Mechanics and Machine Design, Faculty of Mechanical Engineering, Opole University of Technology, Ulica Prószkowska 76, 45-758 Opole, Poland; m.bohm@po.edu.pl

**Keywords:** hemp–lime composite, thermal conductivity, low-energy buildings, specific energy absorption, natural fiber

## Abstract

The main goal of the paper is to calculate the heat conductivity for three experimental hemp–lime composites used for structural construction purposes with the use of the experimental stand inside two compartments. Due to current construction trends, we are constantly searching for eco-friendly materials that have a low carbon footprint. This is the case of the analyzed material, and additional thermographic heat distribution inside the material during a fire resistance test proves that it is also a perfect insulation material, which could be applied in addition of popular isolating materials. This paper presents the results of certain hemp–lime composite studies and the potential for using hemp–lime composite for the structural construction industry. Hemp–lime composite heat transfer coefficient, fire resistance, and bulk density properties are compared to those of other commonly used construction materials. The obtained results show that the material together with supporting beams made of other biodegradable materials can be the perfect alternative for other commonly used construction materials.

## 1. Introduction

The idea of sustainable development in construction, i.e., one that limits the negative impact of buildings on the environment and is user-friendly, is gaining in popularity [1]. Building in an eco-friendly way is becoming common not only among office space developers, who apply for global Leadership in Energy and Environmental Design (LEED) or Building Research Establishment Environmental Assessment Method (BREEAM) certificates, but also among individuals thinking about building a year-round or recreational house [2]. Hemp lime is a material that has a chance to revolutionize the world’s natural construction as it strongly fits into the trend of renewable resources. It has a negative carbon footprint because during its growth, hemp absorbs more carbon dioxide than is used later to make building materials [3,4]. Hemp lime has high thermal insulation, heat accumulation, vapour permeability, is non-flammable, can be used as a fertiliser after demolition and is 100% decomposable [5,6].

The objective of the work is the experimental identification of heat conductivity for three experimental mixtures of hemp–lime composite used for construction purposes analyzed for the use for construction of single-family buildings. Industrial hemp (*Cannabis sativa* L.) has a long history with human civilization and was often found near early nomadic settlements close to streams in well-manured areas [7]. Industrial hemp or *Cannabis sativa* L. is a quick-growing, annual herb with a multitude of uses covering a range of products derived from fiber or oilseed that have been known throughout history [8]. True hemp (industrial hemp) found common application during the 19th century. The sturdy fibers of the plant—well-known and valued for their strength—were used to make ropes for maritime shipping and other industry, as well as paper and textiles [9]. Hemp use has been suppressed in recent times, but due to its usefulness and the ecological advantages in harvesting hemp over cotton and trees, there has been a call for hemp use which is growing worldwide [10].

**Figure 1:**
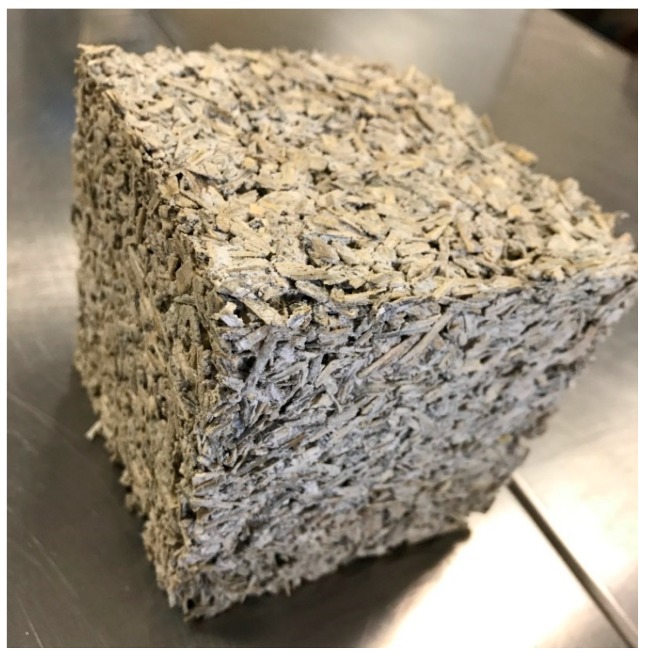
Hemp–lime composite mixture 1.

The hemp–lime composite (Figure 1) consists of water, hemp shiv, lime, and other additives to further improve its properties [5]. Mixing and compressing the components results in a light and appropriately strong material whose structure makes it possible to fill any space or—when applying special formwork—build a complete partition [11,12]. Hemp–lime composite may also be used to make floor tiles or roof and ceiling insulation. Hemp–lime composite shows very poor load-bearing strength and for that reason, it may only be used with specifically designed load-bearing structures or pillars, most typically made of softwood timber [13].

Hemp lime composite debuted in the Polish market just a few years ago and needs extensive testing. The material meets all the requirements for an environmentally-friendly product, a characteristic that plays an ever more significant role [1,14] Some older technologies seem to experience a strong comeback (such as thatched roofs and wooden or adobe homes) owing to their positive impact on human health and comfort and since they utilize natural raw materials [15].

Hemp lime composite owes its excellent thermal conductivity properties to high hemp shiv porosity [16]. Its properties attract even more people both in this country and abroad to launch investigations and promote hemp–lime composite applications in the construction of new or improving thermal insulation performance of the existing buildings [17,18,19,20].

Such composite materials can appear to have variations to their properties due to the existence of structural changes [21]. The properties of the composite are influenced by many factors such as the morphology of the filers, the orientation of the filers, porosity, degree of compaction, the distribution of the filers, and others like gluing [22,23]. Also, some new information regarding nanotubes and fibers in concrete and cementitious materials are worth mentioning [24,25].

Hemp lime composite leaves practically no carbon footprint as hemp shiv, from which it is made, absorbs more CO_2_ from the atmosphere during its lifespan than the amount of CO_2_ released by the manufacturing process. Research showed that one ton of dry hemp sequesters almost 325 kgs of CO_2_ [3].

In hemp–lime composite production, lime is used primarily as a binder, but it also helps inhibit the growth of fungi and mold in the wall. The mixture uses special hydrated lime with an alkaline pH to assure an appropriate biological environment and high vapor permeability of the product. Lime also contributes to better thermal performance and fire resistance properties of hemp–lime composite [26,27].

**Figure 2:**
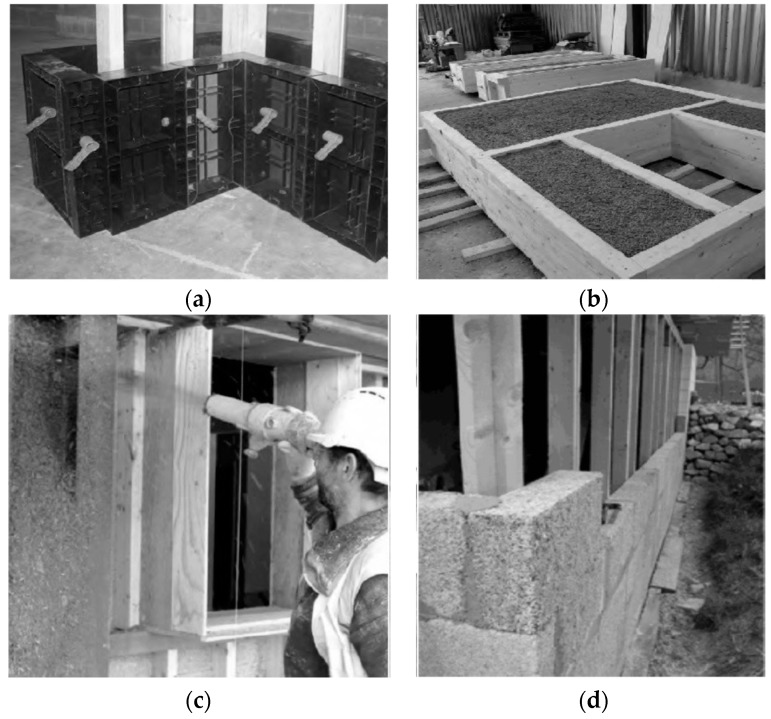
Hemp lime composite techniques: (**a**) casting monolithic walls, (**b**) prefabrication of the entire wall elements, (**c**) spraying, and (**d**) bricklaying [21].

Hemp lime composite has found application in several construction technologies, one of which includes formwork mounted on a wooden frame whose structure corresponds to the design layout of the building walls, floors, and the roof [28]. The fresh mixture is then sprayed into the formwork, compacted, and left to bind. Subsequently, the formwork is removed to allow the partition to dry. Figure 2 shows hemp–lime composite techniques: casting monolithic walls, prefabrication of the entire wall elements, spraying, and bricklaying [29].

## 2. Materials and Methods 

Three different hemp–lime composite mixtures utilizing various binders were studied. Table 1 presents the percentage shares of the components of the tested composites.

Hemp lime composite mixtures study when completed will help define the basic heat conductivity properties of the material, which remain unknown, but which are important for environmentalists and researchers alike. As water plays different roles during the setting and the curing of hemp–lime, and because we needed material with relatively low brittleness and relatively high strength, our experience showed that the chosen in Table 1 water/cement (W/C) ratio was optimal [30,31].

So far, most product information has been supplied by manufacturers who, provide composite performance characteristics, they rather do not specify the detailed composition of the mixtures they use. Thus, comparing the material becomes quite difficult considering the great number of factors affecting the product properties. Some basic factors determining the final properties of the product include the following.

- Hemp shiv type and particle size fractions.
- Type of binder.
- Mixture component proportions.
- Mixing and material application methods.

Despite its ever-growing popularity, the composite properties have not been standardized yet, and that is why it has become necessary to carry out as many tests as possible to establish both the result repeatability and the properties of the product. The objective of our tests consisted of investigating the following properties.

- Bulk density.
- Heat transfer coefficient.
- Fire resistance.

Bulk density is described as a property related to the internal structure of the material; also known as apparent density, it determines the number of properties, such as thermal conductivity, strength, weight, and others. A lime-hemp composite should have a density of 300–500 kg/m^3^ to assure adequate strength and thermal resistance. Apparent density depends mainly on the materials used but also on the density of the compacted mixture. Six samples of all the composite types were tested following a 28-day long maturation process. All the samples were kept in 15 cm × 15 cm × 15 cm containers. Table 2 presents the results obtained once the samples had been removed from the containers: the obtained average sample size of each composite and its volume.

Each container enclosed the same amount of the mixture. Compared to the volume of the wet mixture found in the containers after it had been maturing for 28 days, Mixture 2 is based on hydrated lime with addition of hydraulic lime demonstrated the least shrinkage and volume reduction. Mixture 1 is made of hydraulic lime and only showed the largest volume shrinkage. Table 3 presents the bulk density of the samples following 28-day long maturation of each of the six prepared samples for three composites and their averaged values.

The bulk density of the tested composites seems to be similar; however, Mixture No 3, which also had some amounts of Portland cement, showed a slightly higher density. The composite based on hydraulic lime with some added hydrated lime presented the lowest apparent density, probably due to the calcium oxide turning into calcium hydroxide and thus increasing its volume.

Note that the literature related to hemp–lime composites discusses a lot of other important properties of the material. The compressive strength is one of the more common tests performed, as we can see by the number of papers by Cazacu et al. [32], Kremensas et al. [33], Brzyski et al. [22,34], or Li et al. [35]. As we can see from the information presented in these papers, the compressive strength results obtained in those researches have different values, which vary up to 20% in some cases depending on the mixture type. Another important factor, besides eco-friendliness, seems to be in favor of the hemp–lime composite is its great acoustics properties. We can find many papers related to this issue mostly dealing with the materials acoustic absorption by Kinnane et al. [36], or by Gle et al. [37]. In the last three years, we can also see that a lot of scientists like Bourebrab et al. [38] or Heidari et al. [6] are dealing with the surface coating of hemp–lime composites to increase their resistance to humidity or life cycle.

**Figure 3:**
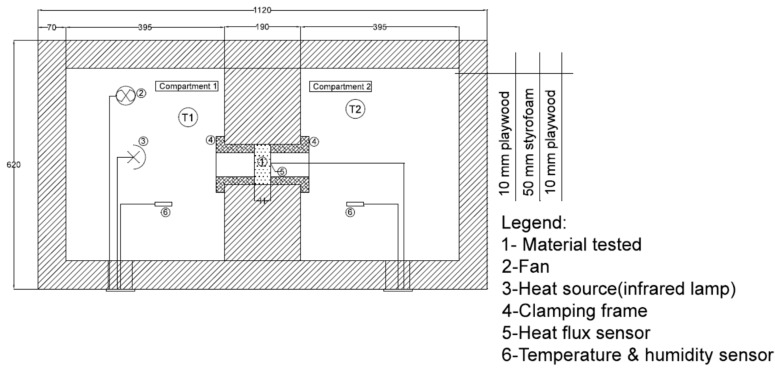
Two-compartment heat box experimental stand in cross section showing its two main compartments.

The main goal of the paper was the calculation of the heat conductivity, which is also related to the value of the heat transfer coefficient of a material. The heat transfer coefficient ”λ” expressed as [W/m*K] describes the insulation properties of the material: the lower the value, the better the insulation parameters of the tested material. To obtain experimental data, the mixtures have been tested inside the two-compartment heat box experimental stand shown in Figure 3.

**Figure 4:**
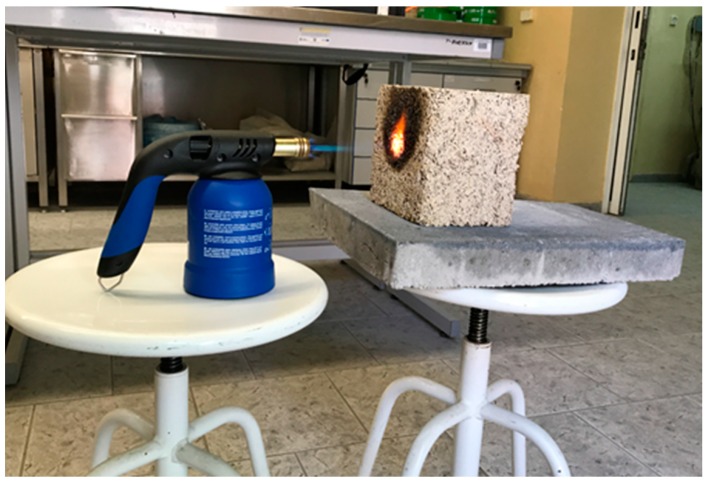
Hemp lime composite flame test, with the tested sample placed at a distance of 10 cm away from the flame source.

Both the inside and outside walls of the box are made of 1.0 cm thick plywood with a 5.0 cm thick Styrofoam insulation layer inside. The box features two compartments separated by a 19.0 cm thick insulated partition, which houses the sample to be tested. The sample is mounted with a clamping frame designed to reduce heat transfer through leaks in the edges caused by the nonuniform structure of the material. A 250W infrared lamp and a fan in Compartment 1 represent the heat source and provide regular air circulation. The study required a sustained 15–20 °C temperature differential between the two compartments be maintained for 2 h. The special insulation of Compartment 2 made it possible to reach the desired measurement stability. Testing a single sample continued for 2 h allowed the temperature and heat flux density to be stabilized. The materials used for testing of the heat conductivity had been weighed and measured to determine their density. Fire resistance of material means its durability, when exposed to high temperatures or flame, with some visual or structural changes if acceptable. The fire resistance test was designed to investigate hemp–lime composite behavior when exposed directly to an open flame and to analyze any ensuing structural changes by calculating potential mass loss. Testing was also performed on three other composite samples measuring 15 × 15 × 15 cm, with a Kemper gas burner of the manufacturer’s maximum rated flame temperature of 1800 °C. The samples, set at a 10 cm distance away from the flame source, were tested at the room temperature of 22.6 °C for 10 min. The entire procedure was recorded with a TESTO 885-1 infrared camera in order to observe the temperature distribution within the material. Figure 4 shows the measuring station.

The primary energy needed by a home erected based on a single design, but in two different technologies, is compared:

- Traditional brick structure: Porotherm, Styrofoam, and wool;
- Natural materials: mainly hemp–lime composite, wattle and daub, and timber.

To this end, energy profiles of the analyzed materials had been prepared using ArCADia TermoCad software (PRO 7 version, Intersoft, Łódź, Poland). ArCADia-TERMOCAD is computer software available in several versions, it is one of the most popular programs on the Polish market designed for preparing energy performance certificates required for construction, modernization, and lease and sale transactions of buildings or premises, as well as for calculating the demand for heat and cooling of rooms and facilities. In ArCADia-TERMOCAD PRO version it is possible to perform energy audits, overhauls, and energy efficiency audits, e.g., for the purpose of obtaining modernization bonus. It can also be used for BREEAM certified calculations. Thanks to a rich database, the user of the program develop the necessary documents in accordance with the legal requirements applicable in Poland. ArCADia-TERMOCAD program in all versions has a built-in, fully functional graphics editor allowing to model the body of the building. The TERMOCADIA editor enables import of drawings in DWG format and import and export of ArCADia BIM system projects. Its main purpose is to perform building designs according to Building Information Modeling (BIM) technology assumptions. In addition to traditional architectural documentation, the program also performs a digital building model [39]. The homes featured an identical mechanical ventilation system capable of recovering roughly 60% of the heat, the same window frames and doors whose heat transfer coefficients complied with the 2020 design standards.

**Figure 5:**
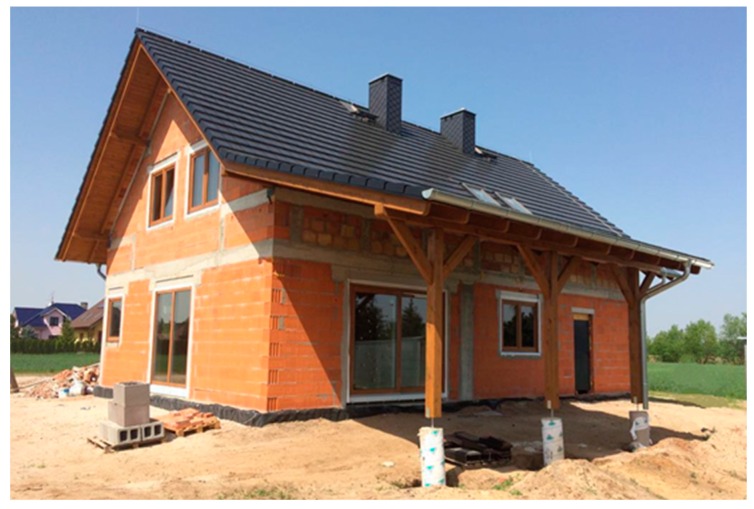
The analyzed building constructed with traditional materials.

The calculations were performed for a single-family two-story home with a loft and a total floor area of ~220.0 m^2^. Building energy performance certificates were issued based on the brick home design, while for our simulation we used the hemp–lime composite home that had been modified by changing its partition structure, i.e., using hemp–lime composite and other natural materials instead. The partitions were fabricated taking into account designers’ and home contractors’ new technology recommendations and guidelines. For our comparative analysis, we selected a new building made of commonly used construction materials as shown in Figure 5. The external walls of the home were made of 24.0 cm wide Porotherm ceramic blocks insulated with 20.0 cm thick Styrofoam (the picture shows the home without the facade). The home features Teriva ceilings and a timber roof truss with complete formwork that has been insulated with 30.0 cm thick Rockwool and covered with ceramic tiles. The “warm installation” method was used to install the balcony doors and windows. The building is heated with a gas-heated condensing boiler.

## 3. Results

Testing was performed while the box was tightly closed. During the tests, the obtained value of the heat transfer coefficient was comparable to that found in the literature and reported by manufacturers. The calculated value of the coefficient was also used later on in the study to determine the power demand of a single-family home constructed with hemp–lime composite. Table 4 presents the results of six samples of the hemp–lime composite that showed the most favorable (lowest) heat transfer coefficient.

Our calculations indicate a traditionally constructed building requires 85.35 kWh/m^2^ × year of nonrenewable primary energy. The energy characteristics provide a lot of vital information about the material and its environmental impact. Table 5 shows selected major highlights of the building energy performance characteristics.

A hemp–lime composite building requires nonrenewable primary energy amounting to 44.81 (kWh/m^2^ × year).

**Figure 6:**
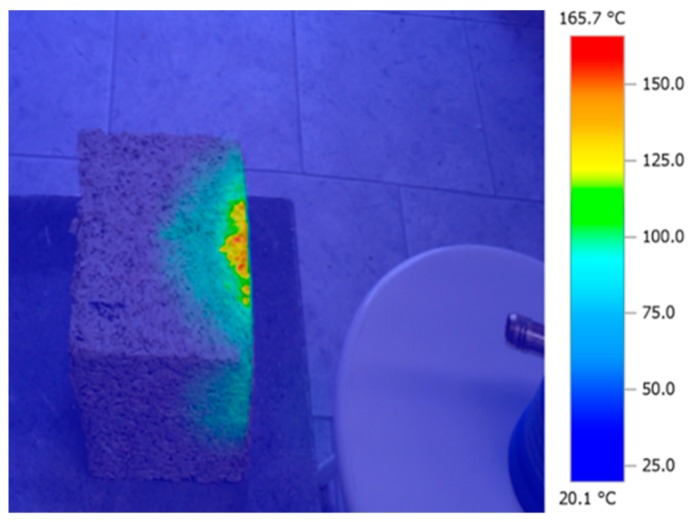
Sample thermal distribution following the flame test.

The thermogram shown in Figure 6 illustrates the temperature distribution within the composite; the picture proves the material has good insulation properties since it does not allow for heat to be transferred to the other side. Manufacturers, suppliers, and various websites promoting hemp–lime often claim fire resistance properties of hemp–lime, but, however, do not provide any actual data [40]. The literature review did not list any research papers pertaining to the fire properties of hemp–lime [41,42]. A clear standard for fire resistance tests is still missing for this type of material samples. The American Society for Testing and Materials (ASTM) did not have a restrictive standard for this type of material. But they are working on a new standard [43]. Nevertheless, fire-resistant tests, under very restrictive conditions, have been performed for the investigated composite sample. The temperature of the flame was 1800 °C during 10 min of testing time. After that time the surface of the sample shown in Figure 6 warmed up to a maximum of 165 °C.

Following a 10 min-long flame test, the composite failed to ignite and showed no tendency to spread the fire. The only effect observed was limited to a certain glow and carbonization of the composite structure, which became considerably weakened around the area exposed to the flame and prone to crumbling. The exposure to flame and oxidization resulted in the composite sample losing some mass. The composite samples had all been carefully weighed using a lab-scale both prior to and following the flame test. Table 6 shows the weight loss results.

The composite based on a hydrated and hydraulic lime binder showed the smallest mass loss of 0.5%, whereas Mixture No 1 lost the most mass compared with other tested mixtures. In summary, results of the hemp–lime composite flame test lead to the conclusion that the material is non-flammable and that using hydrated lime binder may improve the material fire resistance. Following exposure to direct flame, the material structure within the area affected by the high temperature had changed causing significant material weakening due to its increased looseness.

**Figure 7:**
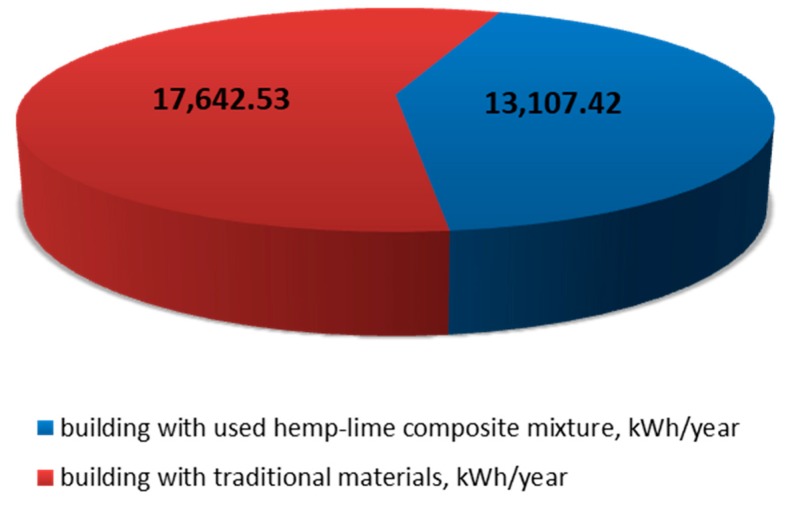
Comparison of total heat losses due to material permeability.

As various materials have been used, losses caused by partition permeability will vary considerably. Figure 7 shows total heat losses due to material permeability.

The building made of natural materials clearly shows much better heat insulation performance compared to a traditional building, owing to lower heat losses attributable to permeability which may reach as much as 4500 kWh per year.

## 4. Discussion

The proposed single-family building structure used hemp–lime composite and other natural materials only. As mentioned before, the same architectural design as the one developed for a building constructed in traditional technology was used. The building energy performance characteristics were then developed for the proposed design using the ArCADia TermoCad software. Commonly available materials and technologies were used to build the hemp–lime composite building. The average value of the coefficient for the composite with the lowest heat transfer coefficient was assumed in Table 7. Table 7 illustrates the structural partition layout required as an atypical construction material was used. For our simulations, we used the results of certain prior hemp–lime composite tests, mainly the heat transfer coefficient of 0.046 W/(m*K) and the 370 kg/m^3^ material density.

The data characterizing the tested material (hemp–lime) were entered into the library of ArCADia-TERMOCAD program, which enabled to calculate the coefficients of penetration of individual building partitions (walls, ceiling, floor, and roof) and, as a result, to determine the energy demand of the whole analyzed building. Next, the obtained results were compared with a building made of traditional building materials used in Poland. The obtained results are presented in Figure 7. The analysis allows to draw a conclusion do that the examined composite can be an alternative to traditional materials, and the calculated energy demand for objects with the same functional arrangement and dimensions is lower for the examined material, which confirms the advisability of using natural materials in single-family buildings.

## 5. Conclusions and Observations

The study was designed to analyze the potential of using natural construction materials for single-family buildings. To perform such an analysis, researchers developed their own natural material to take advantage of the hemp life cycle potential for sustainable construction industry. The most significant benefits of using hemp–lime composite include its potential for use as a non-combustible, renewable and natural raw material with a zero-carbon footprint, and good thermal insulation properties. The lime–hemp composite is very light thanks to its high porosity and low bulk density, which ranges from 300 to 400 kg/m^3^. The parameters give hemp–lime composite very good thermal insulation properties with the thermal conductivity coefficient ranging from 0.038–0.055 (W/m*K), depending on how much the shiv has been compacted and what mixing method was applied. The heat transfer coefficient obtained during the calculations had the value of U_C_ = 0.11 (W/m^2^*K) for external wall and 0.20 (W/m^2^*K) for inside wall with the addition of the hemp–lime composite. The above value makes it possible to erect walls without any additional insulation needed: the hemp–lime composite is a technology that eliminates thermal bridges in the building. The material was also tested for its flame resistance; the sample did not ignite following a 10 min-long exposure to an open 1800 °C flame, because the material contains lime which not only improves its flame resistance but it also protects it from biological degradation corrosion or fungal deterioration [44]. The test results clearly show the use of hydrated lime binder may enhance the material fire resistance characteristics. Utilizing the established properties and parameters of hemp–lime composite, a simulation study was carried out for a home built with hemp–lime composite. A comparison of the power demand characteristics points to a conclusion that a building made of hemp–lime composite will use less energy of each kind, i.e., primary energy Ep, usable energy Eu, and final energy Ef. The only plus of a brick home is that construction materials are easily available at properly qualified contractors. Brick partitions hardly meet the required heat diffusion parameters and the production and application of such materials have been found harmful and detrimental both to the environment andhuman health. Several conclusions may be drawn based on our tests:

- Further development of conventional building materials is not critical for the construction industry since nature offers the best choices it is up to us to use them properly
- Knowledge of hemp–lime as a building material is still at the beginning of the process. In spite of the new research undertaken in this area, there are still no unified standards to ensure the appropriate parameters of a given composite
- A great variability of parameters, such as the conductivity, fire, weather, and biological resistance, of the hemp–lime composite is related to so many factors such as morphology of the fillers, the orientation of the filler particles, porosity, the method and the ratio of compaction, the distribution of the filers and many others
- The authors acknowledge the importance of these factors in terms of hemp–lime structure-related issues. This is a very wide range of interdisciplinary research the authors are in the process of preparing samples of the composites for further investigations.

## Figures and Tables

**Table 1:** Composite mixtures used for testing.

Binder	Composite 1	Composite 2	Composite 3
Hydraulic lime	100%	10%	-
Hydrated lime	-	90%	70%
Portland cement	-	-	30%

**Table 2:** Volume of the samples tested for three composite materials (Mixtures).

Mixture Used	A (cm)	B (cm)	C (cm)	Volume (m^3^)
Mixture 1	15.26	14.90	12.53	0.002849
Mixture 2	15.22	14.92	13.59	0.003086
Mixture 3	15.04	14.69	13.01	0.002874

**Table 3:** Bulk density of the tested mixtures.

Sample No	Mixture 1	Mixture 2	Mixture 3
	Bulk Density of the Samples Following 28-Day Long Maturation (kg/m^3^)		
1	331.28	322.64	370.06
2	329.79	319.56	357.63
3	357.84	338.36	339.14
4	316.40	304.26	337.23
5	308.33	314.99	346.47
6	352.53	354.73	348.17
Average value	333.00	326.00	350.00

**Table 4:** Heat transfer coefficient ”λ” calculations.

Sample No	Left Chamber Temperature (K)	Sample Left Wall Temperature (K)	Right Chamber Temperature (K)	Sample Right Wall Temperature (K)	Heat Flux Density (W/m^2^)	Temperature Gradient (K)	Wall Thickness (m)	λ
								(W/m*K)
1	320.11	318.75	300.03	301.70	14.1	17.05	0.0442	0.037
2	339.00	333.86	301.07	303.02	23.4	30.84	0.0615	0.047
3	340.77	332.23	302.83	307.48	32.4	24.75	0.0343	0.045
4	333.50	333.29	300.18	304.98	41.0	28.31	0.0378	0.055
5	339.05	328.17	302.67	307.28	29.1	20.89	0.0386	0.054
6	331.25	326.53	304.24	307.31	21.9	19.22	0.0346	0.039

**Table 7:** The main partition structure of a hemp–lime composite building.

No	External Wall	d (m)	λ (W/m∙K)	R (m^2^K/W)
	External partition			
1	Clay	0.015	0.850	0.018
2	Concentrated hempcrete	0.400	0.046	8.696
3	Wattle mat	0.010	0.070	0.143
4	Clay	0.015	0.850	0.018
-	Internal partition	-	-	UC = $0.11\;\frac{W}{m^{2}K}$
**No**	**Inside Ceiling**	**d** **(m)**	**λ** **(W/m∙K)**	**R** **(m^2^K/W)**
	Inside partition			
1	Oak fibers lengthwise	0.030	0.400	0.075
2	Pine and spruce fibers crosswise	0.025	0.160	0.156
3	Concentrated hempcrete	0.200	0.046	4.348
4	Pine and spruce fibers crosswise	0.025	0.160	0.156
-	Outside partition	-	-	UC = $\;0.20\;\frac{W}{m^{2}K}$
**No**	**Ground Floor**	**d** **(m)**	**λ** **(W/m∙K)**	**R** **(m^2^K/W)**
	Outside partition			
1	Granulated blast furnace slag, Keramzyt 700	0.300	0.200	1.500
2	Concentrated hempcrete	0.150	0.046	3.261
3	Sand-lime plaster	0.080	0.800	0.100
4	Oak fibers lengthwise	0.025	0.400	0.063
	Inside partition			UC = $0.20\;\frac{W}{m^{2}K}$
**No**	**Roof**	**d** **(m)**	**λ** **(W/m∙K)**	**R** **(m^2^K/W)**
	Outside partition			
1	Wattle slabs	0.350	0.070	5.000
2	Pine and spruce fibers crosswise	0.025	0.160	0.156
3	Hempcrete	0.150	0.044	3.409
4	Pine and spruce fibers crosswise	0.025	0.160	0.156
5	Straw slabs	0.010	0.080	0.125
6	Clay	0.030	0.850	0.035
-	Inside partition	-	-	UC = $0.11\;\frac{W}{m^{2}K}$

**Table 5:** Energy consumption and the environmental impact of a traditional building.

Building Energy Characteristics Evaluation	
Energy characteristics indicators	Analyzed building
Annual usable energy demand	$\mathrm{EU}=52.37\frac{\mathrm{kWh}}{\left(m^{2}\times \mathrm{year}\right)}$
Annual final energy demand	$\mathrm{EK}=71.90\frac{\mathrm{kWh}}{\left(m^{2}\times \mathrm{year}\right)}$
Annual demand for nonrenewable primary energy	$\mathrm{EP}=85.36\frac{\mathrm{kWh}}{\left(m^{2}\times \mathrm{year}\right)}$
CO_2_ emission unit	ECO2 = 0.01497 $\frac{1\;\mathrm{CO}2}{\left(m^{2}+\mathrm{year}\right)}$
The percentage share of renewable energy Resources in annual final energy demand	URER = 0%

**Table 6:** Mass loss calculations following the flame test.

Sample No	Sample Mass before the Test (g)	Sample Mass after the Test (g)	Mass Loss after the Test (g)	Mass Loss Percentage (%)
1	1582.0	1568.4	13.6	0.9
2	1541.4	1533.8	7.6	0.5
3	1598.8	1589.4	9.4	0.6

## References

[1] Bedlivá H., Isaacs N. (2014). Hempcrete—An environmentally friendly material. Adv. Mater. Res..

[2] Radogna D., Mastrolonardo L., Forlani M.C. (2018). Hemp for a healthy and sustainable building in abruzzo. Advances in Intelligent Systems and Computing.

[3] Jami T., Rawtani D., Agrawal Y.K. (2016). Hemp concrete: Carbon-negative construction. Emerg. Mater. Res..

[4] Maalouf C., Ingrao C., Scrucca F., Moussa T., Bourdot A., Tricase C., Presciutti A., Asdrubali F. (2018). An energy and carbon footprint assessment upon the usage of hemp-lime concrete and recycled-PET façades for office facilities in France and Italy. J. Clean. Prod..

[5] Mikulica K., Hela R. (2015). Hempcrete—Cement Composite with Natural Fibres. Adv. Mater. Res..

[6] Heidari M.D., Lawrence M., Blanchet P., Amor B. (2019). Regionalised Life Cycle Assessment of Bio-Based Materials in Construction; the Case of Hemp Shiv Treated with Sol-Gel Coatings. Materials.

[7] Kaiser C., Cassady C., Ernst M. (2015). Industrial Hemp Production. https://www.uky.edu/ccd/sites/www.uky.edu.ccd/files/hempproduction.pdf.

[8] Young E.M. (2005). Revival of Industrial Hemp: A Systematic Analysis of the Current Global Industry to Determine Limitations and Identify Future Potentials within the Concept Of Sustainability. Master’s Thesis.

[9] Allegret S. (2013). The history of hemp. Hemp: Industrial Production and Uses.

[10] Gibson K. (2006). Hemp: A Substance of Hope. J. Ind. Hemp..

[11] Gołębiewski M. (2016). Kompozyty konopno-wapienne (hempcrete). Mater. Bud..

[12] Elfordy S., Lucas F., Tancret F., Scudeller Y., Goudet L. (2008). Mechanical and thermal properties of lime and hemp concrete (“hempcrete”) manufactured by a projection process. Constr. Build. Mater..

[13] Woolley T. (2013). Building physics, natural materials and policy issues. Low Impact Building.

[14] Amziane S., Arnaud L., Challamel N. (2013). Bio-Aggregate-Based Building Materials.

[15] Korjenic A., Petránek V., Zach J., Hroudová J. (2011). Development and performance evaluation of natural thermal-insulation materials composed of renewable resources. Energy Build..

[16] Amziane S., Sonebi M. (2016). Overview on biobased building material made with plant aggregate. RILEM Tech. Lett..

[17] Prabesh K. (2016). Hempcrete Noise Barrier Wall for Highway Noise Insulation: Research & Construction. Bachelor’s Thesis.

[18] Piot A., Béjat T., Jay A., Bessette L., Wurtz E., Barnes-Davin L. (2017). Study of a hempcrete wall exposed to outdoor climate: Effects of the coating. Constr. Build. Mater..

[19] Arnaud L., Gourlay E. (2012). Experimental study of parameters influencing mechanical properties of hemp concretes. Constr. Build. Mater..

[20] Arrigoni A., Pelosato R., Dotelli G. Hempcrete from cradle to grave: The role of carbonatation in the material sustainability. Proceedings of the International Conference on Sustainable Built Environment.

[21] Bolcu D., Stănescu M.M. (2019). The Influence of Non-Uniformities on the Mechanical Behavior of Hemp-Reinforced Composite Materials with a Dammar Matrix. Materials.

[22] Brzyski P., Barnat-Hunek D., Suchorab Z., Łagód G. (2017). Composite Materials Based on Hemp and Flax for Low-Energy Buildings. Materials.

[23] Viel M., Collet F., Pretot S., Lanos C. (2019). Hemp-Straw Composites: Gluing Study and Multi-Physical Characterizations. Materials.

[24] Ramezanianpour A.A., Ghahari S.A., Khazaei A. Feasibility study on production and sustainability of poly propylene fiber reinforced concrete ties based on a value engineering survey. Proceedings of the Sustainable Construction Materials and Technologies.

[25] Ghahari S.A., Ramezanianpour A.M., Esmaeili M. (2016). An Accelerated Test Method of Simultaneous Carbonation and Chloride Ion Ingress: Durability of Silica Fume Concrete in Severe Environments. Adv. Mater. Sci. Eng..

[26] Nguyen T.T., Picandet V., Amziane S., Baley C. (2009). Influence of compactness and hemp hurd characteristics on the mechanical properties of lime and hemp concrete. Eur. J. Environ. Civ. Eng..

[27] Pietruszka B., Gołȩbiewski M., Lisowski P. Characterization of Hemp-Lime Bio-Composite. Proceedings of the IOP Conference Series: Earth and Environmental Science.

[28] Bevan R., Woolley T. Constructing a Low Energy House From Hempcrete and Other Natural Materials. Proceedings of the 11th International Conference on Non-conventional Material Technology (NOCMAT2009).

[29] Bevan R., Woolley T. (2008). Hemp Lime Construction: A Guide to Building. with Hemp lime Composes.

[30] Brocklebank I. (2006). The lime spectrum. Context.

[31] Colinart T., Glouannec P., Chauvelon P. (2012). Influence of the setting process and the formulation on the drying of hemp concrete. Constr. Build. Mater..

[32] Cazacu C., Muntean R., Gălățanu T., Taus D. (2016). Hemp Lime Technology. Bull. Transilv. Univ. Braşov.

[33] Kremensas A., KAIRYTĖ A., Vaitkus S., Vėjelis S., Balčiūnas G. (2019). Mechanical Performance of Biodegradable Thermoplastic Polymer-Based Biocomposite Boards from Hemp Shivs and Corn Starch for the Building Industry. Materials.

[34] Brzyski P., Grudzińska M., Majerek D. (2019). Analysis of the Occurrence of Thermal Bridges in Several Variants of Connections of the Wall and the Ground Floor in Construction Technology with the Use of a Hemp-lime Composite. Materials.

[35] Li Z., Wang X., Wang L. (2006). Properties of hemp fibre reinforced concrete composites. Compos. Part A Appl. Sci. Manuf..

[36] Kinnane O., Reilly A., Grimes J., Pavia S., Walker R. (2016). Acoustic absorption of hemp-lime construction. Constr. Build. Mater..

[37] Glé P., Gourdon E., Arnaud L. (2011). Acoustical properties of materials made of vegetable particles with several scales of porosity. Appl. Acoust..

[38] Bourebrab M., Durand G.G., Taylor A. (2017). Development of Highly Repellent Silica Particles for Protection of Hemp Shiv Used as Insulation Materials. Materials.

[39] INTERsoft ArCADia-TERMOCAD PRO 7. https://www.intersoft.pl/cad/index.php?kup-program-cad=audyt-energetyczny-arcadia-termocad-pro-efektywnosc-energetyczna.

[40] Gregor L. (2014). Performance of Hempcrete Walls Subjected to a Standard Time-temperature Fire Curve. Master’s Thesis.

[41] Daly P. (2011). Hemp lime bio-composite in construction: A study into the performance and application of hemp lime bio-composite as a construction material in Ireland. Proceedings of the PLEA 2011—Architecture and Sustainable Development, 27th International Conference on Passive and Low Energy Architecture.

[42] Amziane S., Arnaud L., Challamel N. (2013). Bio-Aggregate-Based Building Materials: Applications to Hemp Concretes.

[43] ASTM International New Test Methods for Evaluating the Appropriateness/Applicability of Current R-Value and Fire Resistance Test Methods to Testing the Insulative Properties of Hempcrete Insulation Samples. https://www.astm.org/DATABASE.CART/WORKITEMS/WK70549.htm.

[44] Crawford B., Pakpour S., Kazemian N., Klironomos J., Stoeffler K., Rho D., Denault J., Milani A.S. (2017). Effect of Fungal Deterioration on Physical and Mechanical Properties of Hemp and Flax Natural Fiber Composites. Materials.

